# Restates the Law Relating to Malpractice

**Published:** 1898-06

**Authors:** 


### SELECTIONS.

### Restates the Law Relating to Malpractice.

The Court of Appeals of New York says, in the case of Pike vs. Honsinger, March 1, 1898, that the law relating to malpractice is simple and well settled, although not always easy of application. A physician and surgeon, by taking charge of a case, impliedly represents that he possesses, and the law places upon him the duty of possessing, that reasonable degree of learning and skill that is ordinarily possessed by physicians and surgeons in the locality where he practices, and which is ordinarily regarded by those conversant with the employment as necessary to qualify him to engage in the business of practicing medicine and surgery. Upon consenting to treat a patient, it becomes his duty to use reasonable care and diligence in the exercise of his skill and the application of his learning to accomplish the purpose for which he was employed. He is under the further obligation to use his best judgment in exercising his skill and applying his knowledge. The law holds him liable for an injury to his patient resulting from want of the requisite knowledge and skill, or the omission to exercise reasonable care, or the failure to use his best judgment. The rule in relation to learning and skill does not require the surgeon to possess that extraordinary learning and skill which belong only to a few men of rare endowments, but such as is possessed by the average member of the medical profession in good standing. Still, he is bound to keep abreast of the times, and a departure from the approved methods in general use, if it injures the patient, will render him liable, however good his intentions may have been.

The rule of reasonable care and diligence does not require the exercise of the possible care, and, to render a physician and surgeon liable, it is not enough that there has been a less degree of care than some other medical man might have shown, or less than even he himself might have bestowed, but there must be a want of ordinary and reasonable care, leading to a bad result. This includes not only the diagnosis and treatment, but also the giving of proper instructions to his patient in relation to conduct, exercise and the use of an injured limb. The rule requiring him to use his best judgment does not hold him liable for a mere error of judgment, provided he does what he thinks is best after careful examination. His implied engagement with his patient does not guarantee a good result, but he promises by implication to use the skill and learning of the average physician, to exercise reasonable care, and to exert his best judgment in the effort to bring about a good result.

